# Halogenated boroxine increases propensity to apoptosis in leukemia (UT‐7) but not non‐tumor cells *in vitro*

**DOI:** 10.1002/2211-5463.13522

**Published:** 2022-11-22

**Authors:** Maida Hadzic, Yitong Sun, Nikolina Tomic, Eirini Tsirvouli, Martin Kuiper, Lejla Pojskic

**Affiliations:** ^1^ Institute for Genetic Engineering and Biotechnology University of Sarajevo Bosnia and Herzegovina; ^2^ Institute for Biology Norwegian University of Science and Technology Trondheim Norway

**Keywords:** anti‐tumor properties, apoptosis, DEGs, halogenated boroxine, leukemia, pathway enrichment analysis

## Abstract

A hallmark of the development of solid and hematological malignancies is the dysregulation of apoptosis, which leads to an imbalance between cell proliferation, cell survival and death. Halogenated boroxine [K_2_(B_3_O_3_F_4_OH)] (HB) is a derivative of cyclic anhydride of boronic acid, with reproducible anti‐tumor and anti‐proliferative effects in different cell models. Notably, these changes are observed to be more profound in tumor cells than in normal cells. Here, we investigated the underlying mechanisms through an extensive evaluation of (a) deregulated target genes and (b) their interactions and links with main apoptotic pathway genes upon treatment with an optimized concentration of HB. To provide deeper insights into the mechanism of action of HB, we performed identification, visualization, and pathway association of differentially expressed genes (DEGs) involved in regulation of apoptosis among tumor and non‐tumor cells upon HB treatment. We report that HB at a concentration of 0.2 mg·mL^−1^ drives tumor cells to apoptosis, whereas non‐tumor cells are not affected. Comparison of DEG profiles, gene interactions and pathway associations suggests that the HB effect and tumor‐‘selectivity’ can be explained by Bax/Bak‐independent mitochondrial depolarization by ROS generation and TRAIL‐like activation, followed by permanent inhibition of NFκB signaling pathway specifically in tumor cells.

## Graphical abstract

Halogenated boroxine (HB), at a concentration of 0.2 mg·mL^−1^, drives treated tumor cells to apoptosis. Comparison of differentially expressed genes (DEG) profiles, gene interactions and pathway associations suggests that the HB effect and tumor‐‘selectivity’ can be explained as mitochondrial depolarization by ROS generation and TRAIL‐like activation, followed by permanent inhibition of the NFκB signaling pathway in tumor rather than non‐tumor cells.

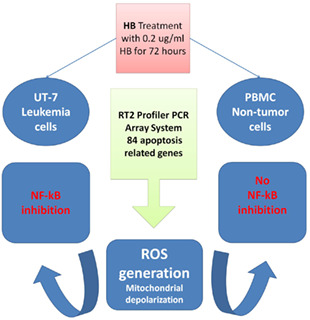

**Abbreviations**
DEGs: differentially expressed genes
HB: halogenated boroxine – K_2_(B_3_O_3_F_4_OH)

A hallmark of the development of solid and hematological malignancies is the dysregulation of apoptosis, which leads to an imbalance between cell proliferation, cell survival and death. Overexpression of apoptosis inhibitors or inactivation of apoptosis promoters has been observed in most of the human cancers [1, 2]. Targeting apoptotic regulators represents a major approach in developing novel anti‐tumor treatments. Due to the genetic heterogeneity of leukemic cells, even within a single patient, there is a growing need for the development of precision treatment strategies [3]. Apoptosis is induced through the activation of one of the two main apoptotic pathways; intrinsic (mitochondrial) and/or extrinsic (via plasma membrane receptors) [4, 5]. Targeted therapies designed to induce apoptosis in leukemia are currently the most promising anti‐leukemia strategies for targeting and eliminating leukemia cells, especially those therapies that invoke relatively minor collateral damage to normal hematopoietic progenitor cells [6, 7]. The regulation of apoptosis by inhibition of proteins from the BCL‐2 family has prompted a search for a new class of anti‐tumor drugs that target anti‐apoptotic members by imitating their natural antagonist (such as BCL‐2 homology proteins and BH3‐only proteins), which are also called BH3 mimetics. These molecules directly activate apoptosis by binding and inhibiting selected anti‐apoptotic members of the BCL‐2 protein family [8, 9].

Halogenated boroxine [K_2_(B_3_O_3_F_4_OH)] (HB) is a derivative of cyclic anhydride of boronic acid [10]. It is patented as a substance with potential antitumor and anti‐proliferative effects [11, 12]. In a series of *in vitro* and *in vivo* studies of the antitumor potential of HB, its tumor‐suppressive effect [13, 14, 15, 16] was observed on different tumor types, in the range of HB treatment concentrations with possible clinical relevance. In a recent study, Hadzic et al. [17] identified that HB negatively affects BCL‐2 expression, but it would be of value to further dissect the mechanism of pro‐apoptotic activity of this compound in leukemia cells by studying the interaction of pathways affected with HB treatment and possible gene regulation switches activated by this compound. Monitoring of apoptosis at the molecular level is possible by analyzing the expression of anti‐ and pro‐apoptotic genes, positive and negative regulators of apoptosis as well as caspase activators and inhibitors, which may help to elucidate some of the main molecular mechanisms of HB negative effects on tumor proliferation and progression. In this study, we aimed to deeply assess a gene expression dataset obtained earlier in work by Hadzic et al. [17] to monitor possible specific effects of HB treatment on leukemia (UT‐7) cells. This involved the analysis of associated pathways involved in apoptosis regulation using different bioinformatics methods to compare the response of tumor and non‐tumor cells upon the most efficient HB treatment (0.2 mg·mL^−1^), to gain new insights into its mechanism of action and to assess possible promising further application.

## Materials and methods

The previously reported pro‐apoptotic effect of HB was investigated by comparing DEG profiles – the corresponding datasets of two reference cell lines: UT‐7 and PBMC (leukemia and control) generated in previous research with special focus on results of treatment with 0.2 mg·mL^−1^ HB that showed the best cytotoxic and cytostatic effects in different experimental settings published so far.

### Gene expression datasets

Relative gene expression quantification data in the form of *C*t values for all target genes (anti‐ and pro‐apoptotic genes, positive and negative apoptosis regulators, as well as genes for caspase activators and inhibitors), were obtained by normalization against standard *GAPDH* expression values (www.qiagen.com/shop/genes‐and‐pathways/data‐analysis‐center‐overview‐page).

### Identification and visualization of DEGs

Gene expression data were converted into a log2FC format associated with an adjusted *P*‐value in each group, scaled by row and clustering_method = ‘complete’. DEGs were called based on log2FC and *P* value. Data points with Benjamini–Hochberg adjustment *P* > 0.05 were considered not significant. The results were processed for display in a heatmap format, using the r package ‘pheatmap’ (version ‘1.0.12’) [18]. The gene expression profiles in groups ‘UT‐7_HB_0.2 mg·mL^−1^’ and ‘PBMC_HB_0.2 mg·mL^−1^’ were further characterized for differential expression patterns with the help of a dumbbell plot, using r package ggplot2 (version ‘3.3.2’) [19, 20]. This involved filtering the 84 genes tested in both samples for *P* < 0.05, leaving 53 significant gene pairs in the comparison.

### GeneMANIA

genemania (http://www.genemania.org; version: 3.6.0) [21] is a user‐friendly web server which contains rich genomics and proteomics data and is used to construct protein–protein interaction networks for functional enrichment analysis. Given the query Gene set 3, functionally related proteins were predicted and enriched pathways were shown.

### Gene ontology (GO) enrichment and KEGG pathway analysis

GO functional enrichment analysis and KEGG pathway analysis were performed on the 53 significant genes in the dumbbell plot, using the r package clusterprofiler (version 3.16.1) [20]. The GO term results include the three categories molecular function (MF), cellular component (CC), and biological process (BP), visualized in the variant chord plot together with gene expression profiles in both tumor and non‐tumor samples via EnrichVisBox (https://www.omicsolution.com/wukong/EnrichVisBox/).

### ClueGO analysis

The cytoscape app cluego [21] was used to analyze and visualize non‐redundant biological terms of a single cluster of genes that have not been previously analyzed in this setting. Comparative analysis of several clusters of genes KEGG pathways is shown in functional groups in the comparison of Gene set 1 and Gene set 2. Gene set 1 was found in leukemia cells as downregulated ‘14 anti‐apoptotic genes’ and Gene set 2 was observed in non‐tumor cells as ‘unaffected genes in normal cells’. Gene set 1 could be an indicator of HB mode of action against leukemia cells since the expression of anti‐apoptotic genes (which are generally increased in tumors) was reduced. Gene set 2 could be an indicator of non‐tumor cell ‘resistance’ to HB activity. cluego (version 2.5.5) was used in combination with cluepedia (version 1.5.5), in cytoscape version 3.7.2 [22].

## Results

Based on the analysis of transcript expression levels of a panel of 84 apoptosis‐associated DEGs, differences in the response of tumor and non‐tumor cells treated with HB were used to gain insight into the affected pathways and differences in response to HB of tumor UT‐7 and non‐tumor PBMC cells.

### Functional differences in deregulated genes following differential treatment

**Fig. 1:**
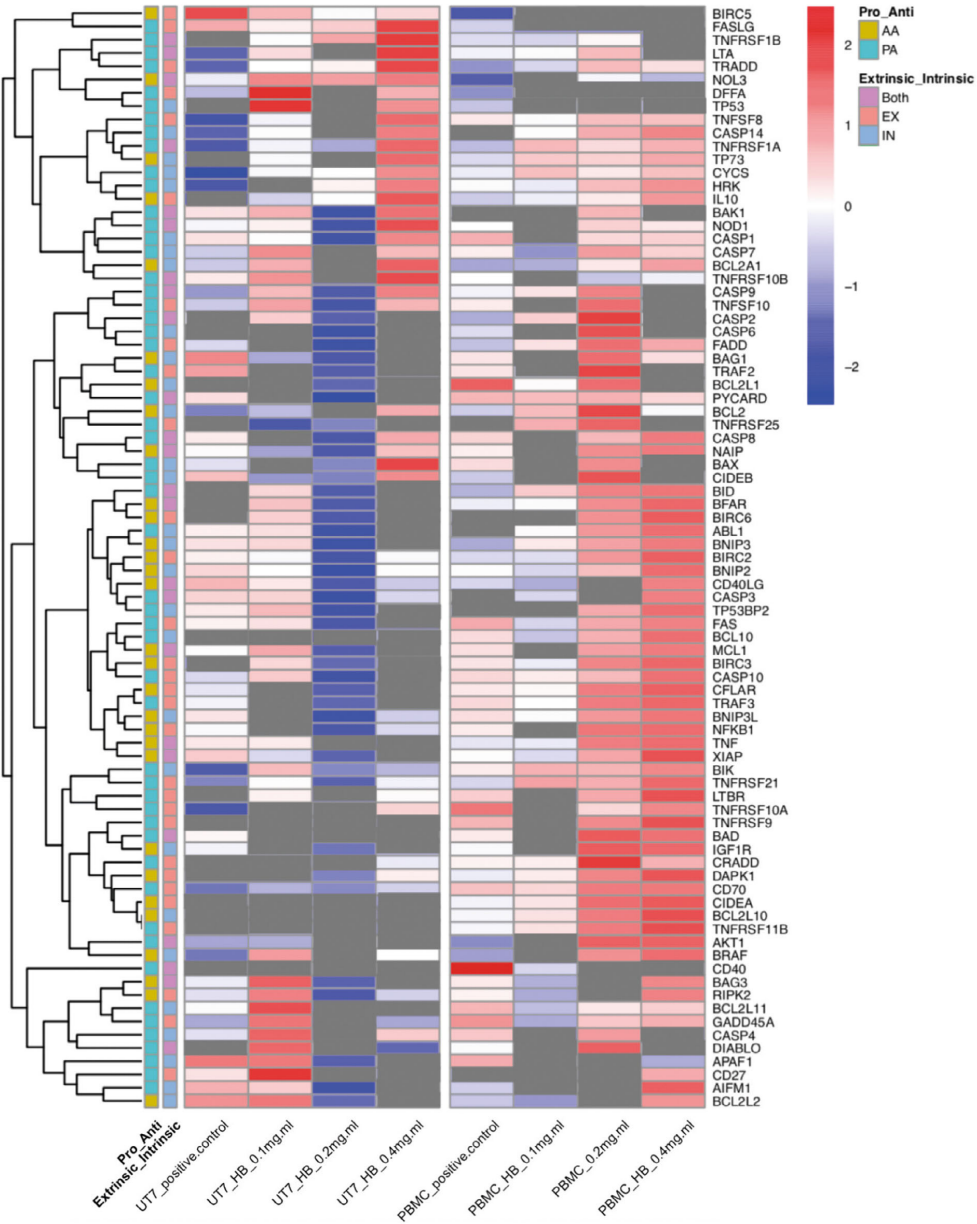
Heatmap representation of expression changes in all 84 genes. Expression changes that do not meet the *P*‐value threshold (0.05) are shown in gray. Colored blocks to the left of the heatmap denote different annotations of the genes. Color codes are explained on the right side of the heatmap: ‘AA’ stands for the anti‐apoptotic genes (gold) and ‘PA’ for the pro‐apoptotic genes (light blue). Each gene is assigned to the intrinsic apoptotic pathway ‘IN’ (blue), extrinsic apoptotic pathway ‘EX’ (red), or involved in both pathways ‘both’ (purple).

**Fig. 2:**
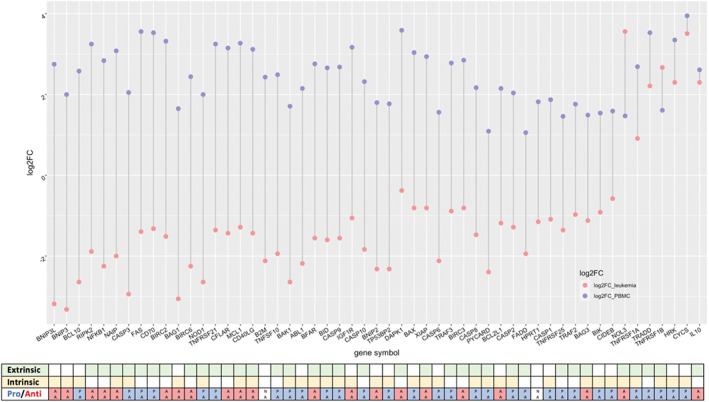
The log2FC separation between UT‐7 leukemia and non‐tumor PBMCs, shown only for 54 differentially expressed genes in HB treatment of 0.2 mg·mL^−1^. In each paired sample, the red dots denote gene expression data from UT‐7 leukemia samples and blue ones from PBMCs, ranked based on the variance of log2FC in UT‐7 and PBMCs, in descending order. Each gene is annotated with extrinsic or intrinsic apoptotic pathways and pro‐ or anti‐apoptotic pathways.

The effects of the HB treatment on DEGs in tumor and non‐tumor cells are compared, over a range of concentrations (Figs 1 and 2). A more detailed classification of these genes to various functional classes show that of the 84 genes present in this panel, 64% can be classified as ‘pro‐apoptotic’ and 36% ‘anti‐apoptotic’ (Fig. 1). Furthermore, 30 of the genes can be assigned to the intrinsic apoptosis pathway, 29 to the extrinsic pathway, and 24 are involved in both.

The transcriptional expression dataset of 84 key genes involved in apoptosis‐related pathways was produced with the RT2 Profiler PCR Array System (Qiagen, Hilden, Germany) as described previously [17]; validated and used as input to obtain graphical presentations of the differential response of targeted genes between two cell‐types treatments. A heat map of the results of the 0.2 mg·mL^−1^ treated cells (Fig. 1) shows that 14 of the 84 anti‐apoptotic key apoptotic genes were differentially expressed between the two cell‐cultures. Notably, 13 anti‐apoptotic genes were downregulated in leukemia cultures, five of which are involved in the extrinsic pathway of apoptosis, three in the intrinsic pathway and six genes are involved in both. However, there is a treatment concentration related optimum observed in PBMCs (non‐tumor cells) that is absent in the UT‐7 cell line (tumor). As the observed pro‐apoptotic effect is highest in tumor cells treated with 0.2 mg·mL^−1^ of HB (Fig. 1), where a large number of genes were downregulated, and because of similar observations in human basal cell carcinoma [13], human GR‐M melanoma [16], mouse mammary adenocarcinoma 4T1, and B16F10 mouse melanoma [14] cell lines, this particular treatment is explored in more detail in our subsequent analyses.

**Fig. 3:**
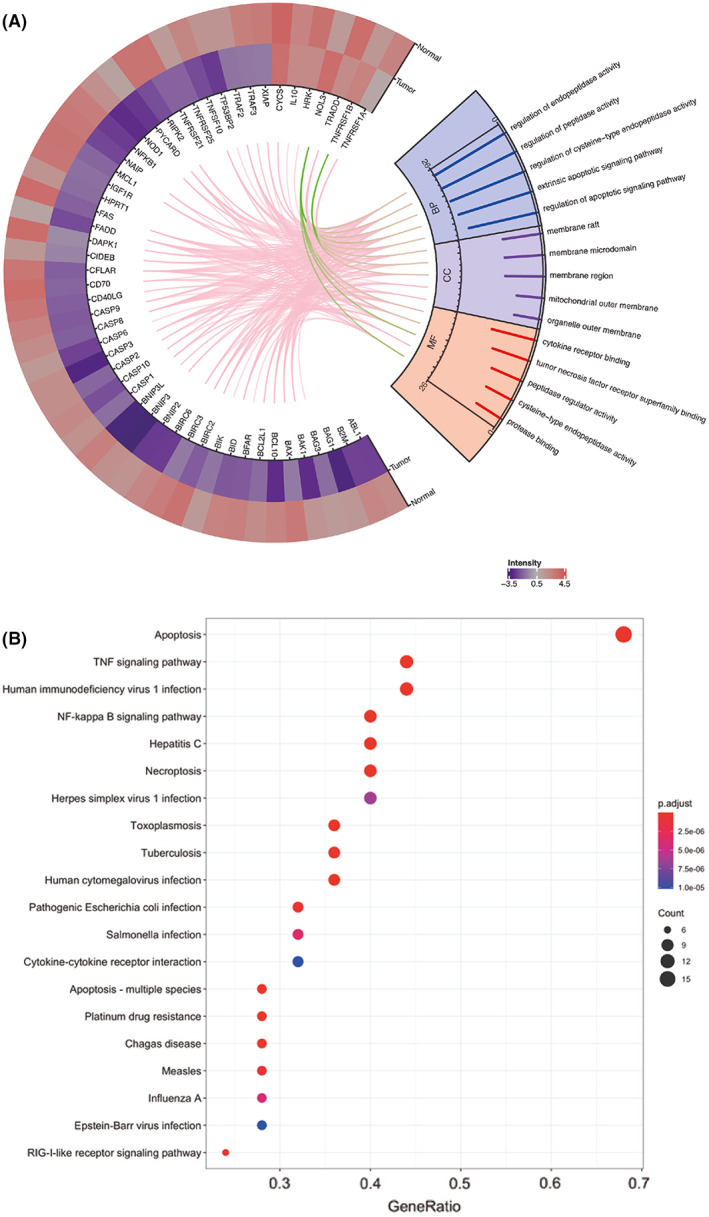
Variant chord plot of DEGs related to apoptosis in non‐tumor and UT‐7 leukemia cells indicates biological process (BP), cellular component (CC) and molecular function (MF) (A) KEGG pathway analysis of DEGs, top 20 shown (B).

A dumbbell plot was constructed to visualize the log2FC (significant fold changes in the 0.2 mg·mL^−1^ HB treatment against relevant negative control) of the UT‐7 and PBMCs lines (Fig. 2). The vast majority of genes show a distinct pattern: downregulation in UT‐7 cells and upregulation in PBMCs. Interestingly, seven genes, namely *NOL3*, *TNFRSF1A*, *TRADD*, *TNFRSF1B*, *HRK*, *CYCS* and *IL10* seem to behave differently as they are all significantly upregulated both in tumor and non‐tumor cells, defined as Gene set 3. Five genes of Gene set 3 belong to the extrinsic apoptosis regulation pathway, which triggers apoptosis via transmembrane receptor‐mediated interactions, as can be observed in Fig. 3A. A genemania (version: 3.6.0) [23] protein–protein interaction network connected Gene set 3 in the inner circle and 20 predicted proteins in the outer circle. This analysis revealed the extrinsic apoptotic signaling pathway as the most significantly enriched, with FDR = 1.69 e^−21^ (Fig. S1). A total of 15 proteins are covered in this pathway, including three genes from Gene set 3, namely *TNFRSF1A*, *NOL3* and *TRADD*. Some of the suggested proteins are also measured in the assay, among them *CFLAR*, *BCL2L1*, and *BIRC2*, which are also found in Gene set 1 as significantly downregulated anti‐apoptotic genes. Other highlighted pathways are regulation of extrinsic apoptotic signaling pathway (FDR = 4.42 e^−17^), extrinsic apoptotic signaling pathway via death domain receptors (FDR = 9.01 e^−15^), and regulation of extrinsic apoptotic signaling pathway via death domain receptors (FDR = 1.72 e^−15^).

### Results of gene ontology (GO) and pathway analysis

Apoptosis pathway focused gene profiling of non‐treated (negative control) compared with HB‐treated UT‐7 leukemia cells and non‐tumor PBMCs was used as an input for further assessments. Functional classification of the 84 genes was performed to identify the biological classes, pathways and pathway modules that were monitored in the analysis of HB treatment responses.

All DEGs in both cell types were imported and GO analysis was performed for sunburst plot construction network representation (Fig. 3A). The top five biological processes (BP) were associated with regulation of peptidase, endopeptidase and cysteine‐type endopeptidase activity, regulation of apoptotic, specifically extrinsic apoptotic signaling. The top five cellular component (CC) terms were membrane raft, microdomain and region as well as mitochondrial and organelle outer membrane. The top five molecular function terms (MF) included cytokine‐receptor binding, tumor necrosis factor receptor superfamily binding, peptidase regulator activity, cysteine‐type endopeptidase activity and protease binding. KEGG pathway analysis (Fig. 3B) shows the top 20 related pathways, with TNF signaling pathway, NF‐kappa B signaling pathway and necroptosis as the most relevant for this study.

**Fig. 4:**
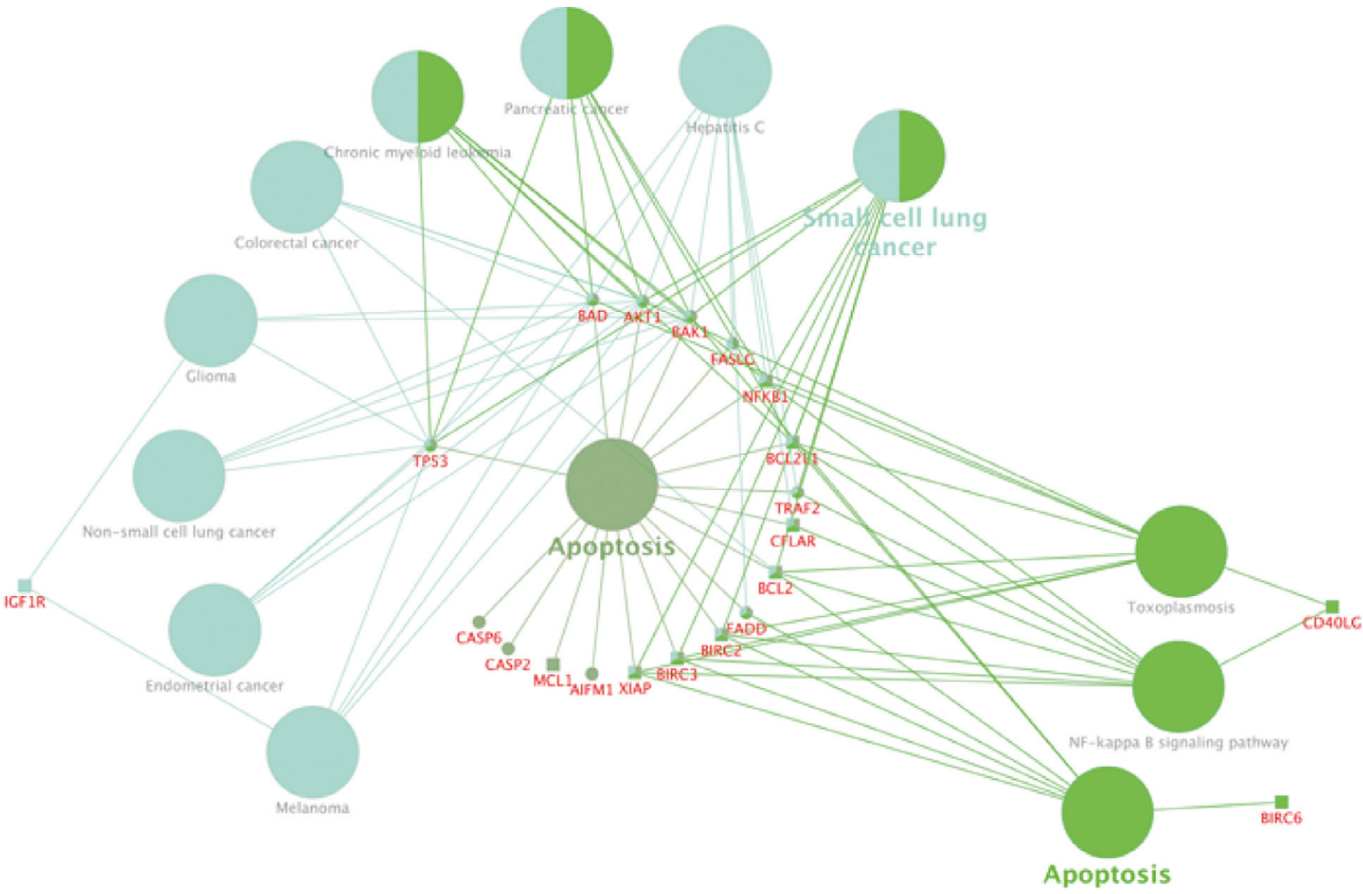
cluego functional comparison analysis results between gene set 1 and gene set 2 against the KEGG pathway database. The pathways enriched are shown as larger ellipses, while genes associated are smaller ones. Genes from gene set 1 shaped as ‘ellipse’ and genes from gene set 2 ‘square’. The *P*‐value threshold is set to be 0.05 and the pathway network connectivity (kappa score) is set to be 0.4. Color coding: blue lines suggest links to anti‐apoptotic and green lines suggest links to pro‐apoptotic pathways.

One of the most interesting sets of genes was named the ‘14 anti‐apoptotic genes’ (Gene set 1), that was found to be downregulated with HB treatment in both cell types regardless of treatment concentration: *BAG3*, *BCL2*, *BCL2L1*, *BFAR*, *BIRC2*, *BIRC3*, *BIRC6*, *CD40LG*, *CFLAR*, *IGF1R*, *MCL1*, *NAIP*, *NFKB1* and *XIAP*. A second set comprised ‘unaffected genes in normal cells’ (Gene set 2), with *AIFM1*, *AKT1*, *BAD*, *BAG1*, *BAK1*, *BCL2L2*, *CASP2*, *CASP6*, *CD27*, *CIDEB*, *FADD*, *FASLG*, *TNFRSF25*, *TP53* and *TRAF2*, and was used to explore the basis for resistance of healthy cells (PBMCs) to antitumor and anti‐proliferative effects of HB. The ClueGO functional analysis results of the comparison between the ‘14 anti‐apoptotic genes’ gene set and the ‘unaffected genes in normal cells’ gene set against the KEGG pathway database show that deregulation of selected anti‐apoptotic and pro‐apoptotic genes concomitantly affects several pathways directly associated with promotion of programmed cell death following HB treatment. The largest number of target genes deregulated by HB treatment is engaged in regulation of apoptosis via three specific pathways: NF‐kappa B signaling (eight genes directly engaged in apoptosis associated pathways), toxoplasmosis (eight genes associated with cellular immune response), and direct apoptosis (seven genes including mediation via BIRC6 family of proteins; Fig. 4).

For a further assessment of specific functional mechanisms explaining the HB effects on gene deregulation at the transcriptional level, we used Reactome (Tables S1 and S2) and panther (Tables S3 and S4) [24, 25]. This analysis revealed shared pathway hits linked to cytotoxic and apoptosis related processes, which is of particular interest in UT‐7 leukemia cells upon HB treatment. Common annotations among the 20 most significant pathways in leukemia cells included regulation of intrinsic and extrinsic apoptotic pathways, regulation of other types of cell death such as anoikis, necrosis or necroptosis, NFκB activity mediation, regulation of endopeptidase activity, and protein poly‐ubiquitination.

## Discussion

The anti‐proliferative effect of HB in tumors and not in non‐tumor cells has been observed previously [15], yet the mechanism of this effect was not explored in any detail. We used a gene expression dataset targeting genes that are implicated in apoptosis, to identify the genes deregulated by HB in leukemia cell line (UT‐7) and non‐tumor cells (PBMCs) that could explain for differences in cytotoxicity observed between tumor and healthy cells. As this gene set comprises mainly apoptosis‐related genes, we focused on comparing the specific pro‐apoptotic mechanism triggered in UT‐7 and PBMC cells, and corresponding signaling pathways to elucidate the underlying mechanism of stronger cytotoxic HB action in tumor cells.

The most remarkable feature of cancer is the ability of tumor cells to escape apoptosis through shifting the balance of pro‐apoptotic and anti‐apoptotic proteins, reduced expression of caspase, and a loss of death receptor signaling [26]. Anti‐apoptotic genes are stably expressed in tumors, constituting one of the basic mechanisms for avoiding apoptosis and contributing to dysregulation of cell proliferation [26]. Compounds that impinge on this mechanism, i.e. Bcl‐2 antagonists or BH3 mimetics, may prove to have useful anti‐cancer properties. According to DEGs representation (Fig. 1) the most anti‐apoptotic genes were downregulated in UT‐7 cells in HB 0.2 mg·mL^−1^ treatment suggesting triggering of apoptosis and necrosis only in leukemia (Figs 1 and 2). The BCL‐2 and MCL1 joint downregulation is previously confirmed in AML cell lines as a mechanism of their synergistic pro‐apoptotic effect that underpins the anti‐leukemic response [27]. These data suggest that HB induces a pro‐apoptotic cascade of events by affecting pro‐apoptotic pathways rather than to have a direct antagonistic effect to Bcl‐2. In another recent study on GR‐M human melanoma [16], more than 30 tumor associated genes were found to be deregulated in cancer cells upon treatment with HB. Conversely, in non‐tumor PBMCs, all anti‐apoptotic genes were upregulated, which may be the most common cellular response to stress stimuli as it provides them the ability to repair potential damage i.e. from ROS generation [28], by activation of inflammation pathways rather than entering apoptosis [29]. As described previously [17], similar observations have been reported for non‐tumor control cells in other experiments.

In this work, we showed that UT‐7 and PBMC cultures react differently to treatment with HB. In leukemia cells grown *in vitro*, treatment with HB evidently induces the TNFα [26] regulated pro‐apoptotic cascade, whilst in PBMC a pro‐inflammatory process is induced with notable association of anti‐apoptotic factors (c‐FLIP, caspase‐8 and ‐10), which forms the apoptosis inhibitory complex (AIC). Upregulation of anti‐apoptotic factors was observed less in UT‐7 cells, which explains why pro‐apoptotic events in these two types of cells are not of the same intensity and nature (Table S4). According to the results of log2FC separation between UT‐7 and PBMCs (Fig. 2) for DEGs after HB 0.2 mg·mL^−1^ treatment, seven genes (*NOL3*, *TNFRSF1A*, *TNFRSF1B*, *TRADD*, *HRK*, *CYCS* and *IL10*) were upregulated in both cell types. These specific responses indicate that at least parts of the apoptotic triggers are common to both cell types. It could be explained that HB triggers apoptosis in both cells, possibly in the same way via the death receptor extracellular signaling by activation of TNFα signaling cascade [26], but with different signal processing and final outcome in tumor and normal cell types. In PBMC, this signal is transmitted but within 72 h it is stabilized possibly by intrinsic anti‐apoptotic mechanism activation without committing cells to apoptosis. This finding is in line with previously observed effects of HB treatment compared with positive apoptosis control treatment in UT‐7 cells using other cytogenetic methods of evaluation [17]. The observed difference in response between the two cell types might be related to the adaptive response of PBMCs to induced cellular stress, resulting in a transient initiation of the process of apoptosis, without a full commitment [28, 29]. The activation of genes associated with the oxidative damage pathways (cellular responses to induced stress; Table S4) only in PBMCs further supports the idea of a differential response to HB. In extension to this, tumor cells (UT‐7) are more prone to pro‐apoptotic effects of HB, with several activated genes suggesting that other forms of cell death (necrosis, necroptosis, anoikis) in UT‐7 cells are also possible (Table S3) and have been first observed here. Association with those types of cell death could provide additional insight into the HB mechanism of action considering their targeting in tumors [30, 31].

An important subset of responder genes (Gene set 1‐ downregulated anti‐apoptotic genes in leukemia) is associated with the NF‐κB signaling pathway (Figs 3B and 4; Table S1), which plays a fundamental role in the development of AML and represents an attractive target for therapeutic intervention [32, 33, 34]. In fact, new NF‐κB signaling inhibitors offer a promising strategy for the development of candidate drugs in antitumor therapy [35]. NFκB inhibition is associated with the proteasome inhibition by bortezomib (Velcade, Millennium Pharmaceuticals, Cambridge, MA, USA), an antitumor agent chemically similar to HB. Bortezomib shows tumor‐selective toxicity and its mechanism of toxicity is based on inhibition of NFκB [36]. Studies of apoptosis induction in other tumor cell types suggest the possibility of Bax/Bak‐independent apoptosis induction with TRAIL agonists, which bind to the tumor cell surface [37] and may lead to a tumor cell sensitization by ROS generation and mitochondrial depolarization as a way to override anti‐apoptotic mechanisms that are upstream of the mitochondrial apoptosis cascade. As boron‐containing compounds (BCCs) are widely used for tumor sensitization to treatment, the observed HB selectivity to tumors could be additionally explained by its boron constituent [38, 39, 40]. According to DEGs and GO analysis (Fig. 3A), the most prominent biological processes related to peptidase and endopeptidase activity implicate HB as possible peptidase regulator and protease binding moderator. This kind of HB activity might be expected because it was previously reported that HB interferes with different enzyme activities [41, 42, 43, 44]. In addition, statistically significant inhibition of anti‐apoptotic BCL‐2 protein by HB 0.2 and 0.4 mg·mL^−1^ treatments was confirmed in UT‐7 leukemia only [17]. Positive regulation of protein polyubiquitination was also observed among the significantly affected biological processes in UT‐7 leukemia (Table S3). It seems that HB as a new BCC, might have a potential to inhibit formation of key cellular proteins involved in anti‐apoptotic processes. However, the relationship between NFκB activity inhibition and peptidase activity of HB is based on apoptotic events, which are also suggested by some GO Cellular Component terms (membrane region and mitochondrial membrane inclusion; Fig. 3A). Changes in mitochondrial membrane permeability and potential are one of the first signs of intrinsic apoptotic pathway events [45]. Additionally, the GO enrichment of extrinsic apoptotic signaling supports the induction of apoptosis in this way, which is also related to transmembrane death domain factors mediation and seven observed upregulated genes in both cells. A genemania protein–protein interaction network connected genes from the Gene set 3 with 20 predicted proteins and the analysis revealed the extrinsic apoptotic signaling pathway as the most significant enriched function. It also highlighted several additional extrinsic apoptosis related pathways, giving overall deeper insight into functional relationships of HB treatment with induction of apoptosis.

All pathway classification results suggest that HB has an evident pro‐apoptotic effect in UT‐7 leukemia but not in non‐tumor PBMCs. This apparent selectivity of treatment effect on tumor cells is caused by apoptosis induction via deregulation of genes engaged mainly in pro‐apoptotic cellular processes and inhibition of anti‐apoptotic regulators highly expressed in tumors. Since similar HB‐selective responses have been observed both *in vitro* and *in vivo* with a calcium ion‐dependent effect [15], one of the possible explanations of the cancer specific effect of HB could be its effect on ion balance and cell membrane depolarization leading to apoptosis. In this study, we found that the HB effect in UT‐7 leukemia is realized through the extrinsic apoptosis induction via death domain receptors, ROS generation and concomitant mitochondrial membrane depolarization followed by intrinsic apoptosis activation. Extended deregulation of genes engaged with NF‐κB and peptidase activity mediation ensures apoptosis signaling persistence in tumor cells and not in non‐tumor (PBMC) cells.

## Conclusions

Searching for drugs that could induce apoptosis in tumor cells is a main trend in current anticancer research. In this work, we showed that HB, especially at a concentration of 0.2 mg·mL^−1^, initiates a process of tumor cells sensitization to pro‐apoptotic activation, probably via the Bax/Bak‐independent mitochondrial depolarization by ROS generation and TRAIL‐like activation, followed by inhibition of NFκB signaling pathway. This is supported by reproducible HB effects observed in different cell types and extensive bioinformatics analysis of treatment induced DEGs as markers, their functional interactions and pathway enrichment analysis and warrants further functional studies for therapeutic use of HB.

## Conflict of interest

The authors declare no conflict of interest.

## Author contributions

LP and MK conceived and designed the project; MH, NT and YS acquired the data; ET, YS, NT and MH analyzed and interpreted the data and ALL participated in manuscript drafting and revisions.

## Supporting information


**Fig. S1.** Protein–protein interaction network of seven tail genes (Gene set 3).
**Table S1.** The 20 most significant Reactome pathways of 14 anti‐apoptotic genes in leukemia cells, sorted by *P*‐value.
**Table S2.** The 20 most significant *Reactome* pathways of unaffected genes in non‐tumor PBMCs, sorted by *P*‐value.
**Table S3.** The 20 most significant panther (Version 15.0) BP results of 14 anti‐apoptotic genes in leukemia cells, sorted by *P*‐value.
**Table S4.** The 20 most significant panther (Version 15.0) BP results of unaffected genes in non‐tumor PBMCs, sorted by *P*‐value.

## Data Availability

The data that support the findings of this study are available from the corresponding author [martin.kuiper@ntnu.no] upon reasonable request.
