# Supplementary material for: Halogenated boroxine increases propensity to apoptosis in leukemia (UT‐7) but not non‐tumor cells in vitro
Source: FEBS Open Bio. 2022 Nov 22;13(1):143–53. doi: 10.1002/2211-5463.13522 (PMC9811610; doi:10.1002/2211-5463.13522)
Supplement: Supplementary file 1 — Fig. S1. Protein–protein interaction network of seven tail genes (Gene set 3). Table S1. The 20 most significant Reactome pathways of 14 anti‐apoptotic genes in leukemia cells, sorted by P‐value. Table S2. The 20 most significant Reactome pathways of unaffected genes in non‐tumor PBMCs, sorted by P‐value. Table S3. The 20 most significant panther (Version 15.0) BP results of 14 anti‐apoptotic genes in leukemia cells, sorted by P‐value. Table S4. The 20 most significant panther (Version 15.0) BP results of unaffected genes in non‐tumor PBMCs, sorted by P‐value. [file FEB4-13-143-s001.docx]

**Halogenated boroxine increases propensity to apoptosis in leukemia (UT-7)**

**but not non-tumor cells *in vitro***

Maida Hadzic^1*^, Yitong Sun^2*^, Nikolina Tomic^1^, Eirini Tsirvouli^2^, Martin Kuiper^2^ and Lejla Pojskic^1^

*
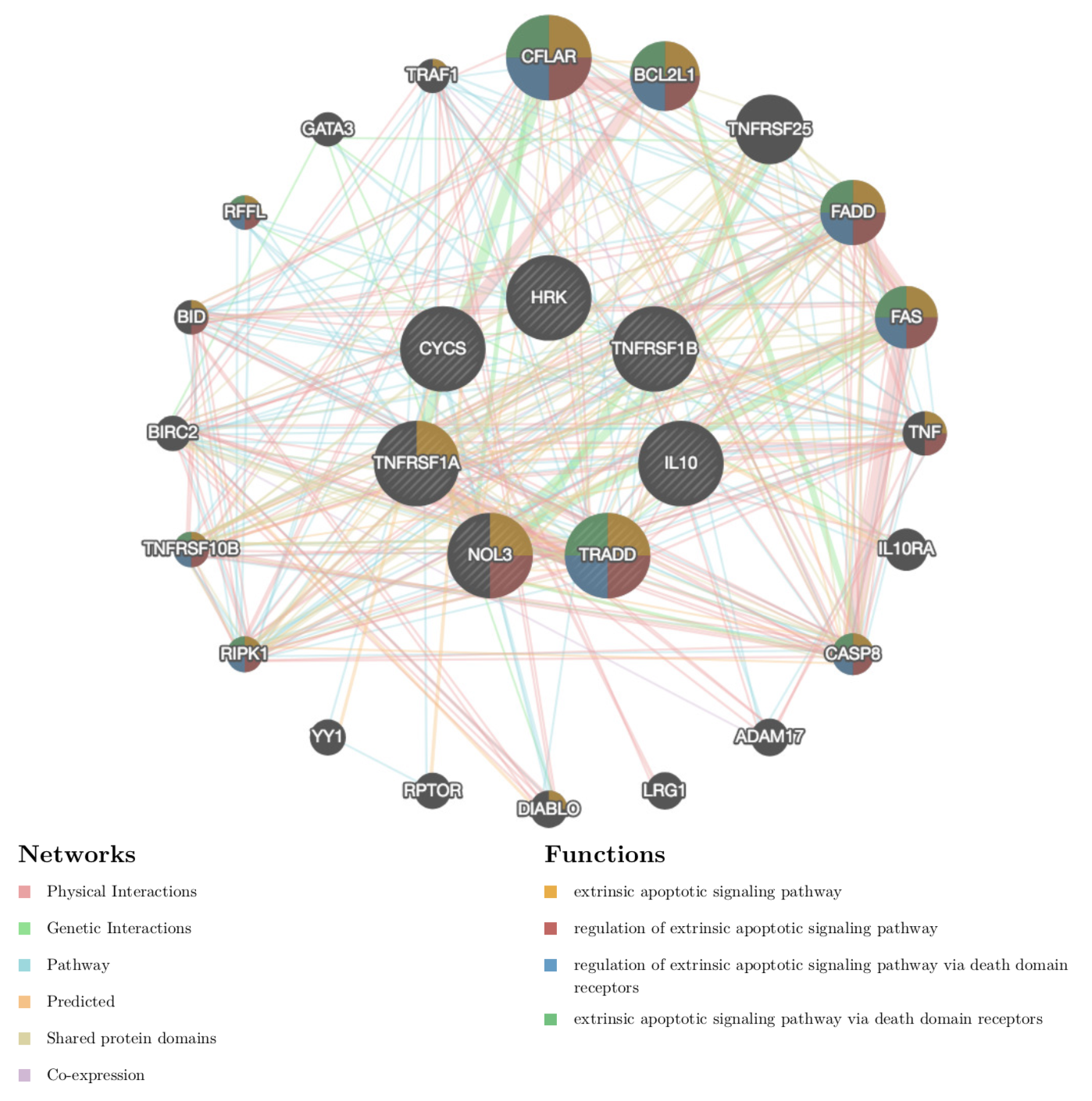
*

*Supplementary Figure S1.* Protein-protein interaction network of 7 tail genes (Gene set 3).

*Supplementary Table S1.* The 20 most significant Reactome pathways of 14 anti-apoptotic genes in leukemia cells, sorted by p-value.


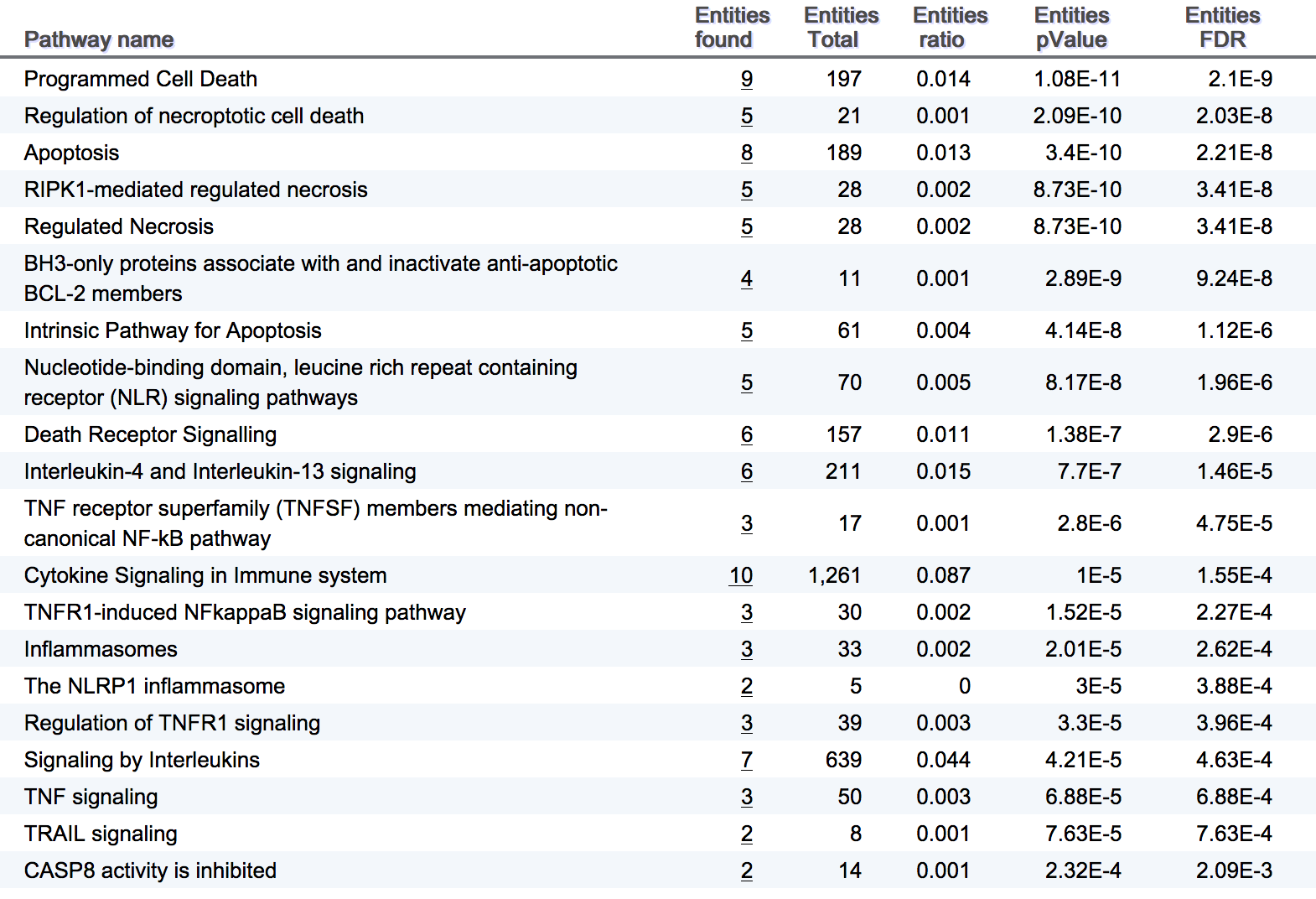


*Supplementary Table S2.* The 20 most significant *Reactome* pathways of unaffected genes in non-tumor PBMCs, sorted by p-value.


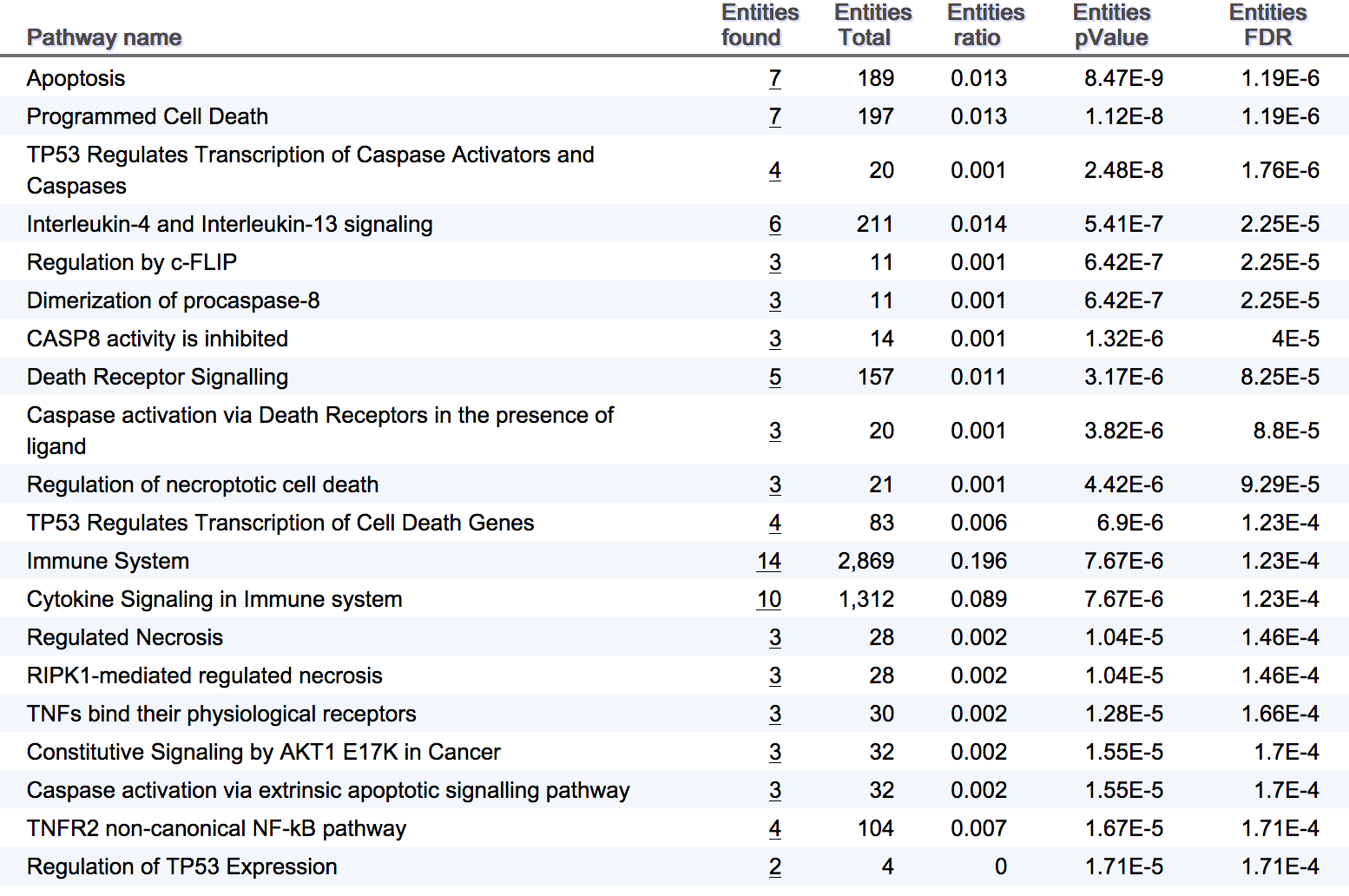


*Supplementary Table S3.* The 20 most significant *Panther* (Version 15.0) BP results of 14 anti-apoptotic genes in leukemia cells, sorted by p-value.

|  | **GO biological process complete** |
| --- | --- |
| 1 | regulation of nucleotide-binding oligomerization domain containing signaling pathway |
| 2 | inhibition of cysteine-type endopeptidase activity |
| 3 | zymogen inhibition |
| 4 | negative regulation of necroptotic process |
| 5 | negative regulation of programmed necrotic cell death |
| 6 | negative regulation of necrotic cell death |
| 7 | regulation of necroptotic process |
| 8 | negative regulation of anoikis |
| 9 | regulation of programmed necrotic cell death |
| 10 | positive regulation of protein polyubiquitination |
| 11 | signal transduction in absence of ligand |
| 12 | extrinsic apoptotic signaling pathway in absence of ligand |
| 13 | regulation of anoikis |
| 14 | regulation of necrotic cell death |
| 15 | regulation of RIG-I signaling pathway |
| 16 | release of cytochrome c from mitochondria |
| 17 | negative regulation of protein processing |
| 18 | negative regulation of protein maturation |
| 19 | regulation of viral-induced cytoplasmic pattern recognition receptor signaling pathway |
| 20 | regulation of protein polyubiquitination |

*Supplementary Table S4.* The 20 most significant *Panther* (Version 15.0) BP results of unaffected genes in non-tumor PBMCs, sorted by p-value.

|  | **GO biological process complete** |
| --- | --- |
| 1 | necroptotic signaling pathway |
| 2 | positive regulation of I-kappaB phosphorylation |
| 3 | regulation of I-kappaB phosphorylation |
| 4 | death-inducing signaling complex assembly |
| 5 | programmed cell death involved in cell development |
| 6 | activation of cysteine-type endopeptidase activity |
| 7 | positive regulation of mitochondrial membrane potential |
| 8 | positive regulation of B cell differentiation |
| 9 | programmed necrotic cell death |
| 10 | necrotic cell death |
| 11 | T cell apoptotic process |
| 12 | necroptotic process |
| 13 | cellular response to nitric oxide |
| 14 | signal transduction in absence of ligand |
| 15 | extrinsic apoptotic signaling pathway in absence of ligand |
| 16 | positive regulation of membrane potential |
| 17 | positive regulation of release of cytochrome c from mitochondria |
| 18 | cellular response to reactive nitrogen species |
| 19 | lymphocyte apoptotic process |
| 20 | regulation of release of cytochrome c from mitochondria |
